# Home mortgage discrimination and incidence of triple-negative and Luminal A breast cancer among non-Hispanic Black and non-Hispanic White females in California, 2006–2015

**DOI:** 10.1007/s10552-022-01557-y

**Published:** 2022-02-03

**Authors:** Eli K. Michaels, Alison J. Canchola, Kirsten M. M. Beyer, Yuhong Zhou, Salma Shariff-Marco, Scarlett L. Gomez

**Affiliations:** 1 Division of Epidemiology, Berkeley School of Public Health, University of California, 2121 Berkeley Way #5302, Berkeley, CA 94720-7360 USA; 2 Department of Epidemiology and Biostatistics, University of California, San Francisco, CA USA; 3 Greater Bay Area Cancer Registry, San Francisco, CA USA; 4 Division of Epidemiology and Social Sciences, Institute for Health and Equity, Medical College of Wisconsin, Milwaukee, WI USA; 5 Helen Diller Family Comprehensive Cancer Center, University of California, San Francisco, CA USA

**Keywords:** Triple negative breast cancer, TNBC, Incidence rates, Social environment, Neighborhood context, Institutional racism

## Abstract

**Purpose:**

In the United States, Black females are burdened by more aggressive subtypes and increased mortality from breast cancer compared to non-Hispanic (NH) White females. Institutional racism may contribute to these inequities. We aimed to characterize the association between home mortgage discrimination, a novel measure of institutional racism, and incidence of Luminal A and triple-negative breast cancer (TNBC) subtypes among NH Black and NH White females in California metropolitan areas.

**Methods:**

We merged data from the California Cancer Registry on females aged 20 + diagnosed with primary invasive breast cancer between 2006 and 2015 with a census tract-level index of home mortgage lending bias measuring the odds of mortgage loan denial for Black versus White applicants, generated from the 2007–2013 Home Mortgage Disclosure Act database. Poisson regression estimated cross-sectional associations of census tract-level racial bias in mortgage lending with race/ethnicity- and Luminal A and TNBC-specific incidence rate ratios, adjusting for neighborhood confounders.

**Results:**

We identified *n* = 102,853 cases of Luminal A and *n* = 15,528 cases of TNBC over the study period. Compared to NH Whites, NH Black females had higher rates of TNBC, lower rates of Luminal A breast cancer, and lived in census tracts with less racial bias in home mortgage lending. There was no evidence of association between neighborhood racial bias in mortgage lending at the time of diagnosis and either subtype among either racial/ethnic group.

**Conclusion:**

Future research should incorporate residential history data with measures of institutional racism to improve estimation and inform policy interventions.

## Introduction

Breast cancer is the most commonly diagnosed cancer and the second-leading cause of death from cancer among females in the United States. Considerable inequities in breast cancer severity and mortality have been documented between Black and non-Hispanic (NH) White females. Although incidence rates have declined among most racial/ethnic groups, they have steadily increased among Black females, to a national level that is similar to that of NH White females, who have historically had the highest incidence rates [1, 2]. Black females are also twice as likely as NH White females to be diagnosed with triple-negative breast cancer (TNBC) [3–5], which is more aggressive and less responsive to current treatments than the hormone receptor-positive (HR +) subtypes, such as Luminal A [3, 4, 6]. NH White females, in contrast, have the highest incidence of Luminal A breast cancer, the most common subtype with the most favorable prognosis [3, 4]. Once diagnosed, Black females have roughly 42% higher mortality from breast cancer compared to NH Whites [7, 8].

There is increasing recognition of the role of institutional racism, defined as “differential access to the goods, services, and opportunities of society by race” [9] in the production of racial inequities along the breast cancer continuum [2, 10–13]. Housing discrimination is one primary form of institutional racism in the United States [14–17]. Although explicit discrimination has been illegal since the passage of the Fair Housing Act of 1968, covert forms of home mortgage discrimination against Black Americans persist, contributing to patterns of racial segregation that have remained strikingly stable since the 1860s [14–17]. Emerging evidence documents associations between racial residential segregation and breast cancer incidence and mortality [11, 18–20]. Importantly, however, racial residential segregation is a proxy measure that captures the consequence of institutional racial discrimination, not the discrimination itself [21]. In order to hold institutions and decision-makers accountable and inform policy change, there is a pressing need to rigorously interrogate the direct effects of discriminatory practices, such as in home mortgage lending practices, on breast cancer outcomes and inequities [22].

Publicly available data from the Home Mortgage Disclosure Act (HMDA) provide an opportunity for researchers to document the extent of contemporary housing discrimination across communities and examine its associations with health inequities [10, 21, 23–25]. The HMDA was enacted by congress in 1975 “to make lending practices transparent, ethical, responsive to community needs” [26]. The Act requires lenders to report annual data on the location of housing loans and whether the loan was approved or denied, as well as demographic characteristics of the applicants [26]. Using these data, researchers can quantify racial bias in home mortgage lending, or the degree to which Black applicants are disproportionately denied loans relative to White applicants, adjusting for income and other relevant characteristics [10, 21, 23–25].

Previous research has documented associations between home mortgage discrimination and health outcomes [21, 23, 24], including breast cancer mortality [10, 25]. However, no studies, to our knowledge, have explored the relationship between housing discrimination and breast cancer incidence. This is an important topic because identifying risk factors for the development of cancer can inform primary prevention efforts. Neighborhoods where Black families face institutionalized discrimination and systemic exclusion could potentially represent toxic social environments which may be associated with greater breast cancer risk and the development of more aggressive subtypes [12, 27, 28].

Breast cancer is a heterogeneous disease and subtypes defined by hormone-receptor (HR) biomarkers and human epidermal growth factor receptor (HER2Neu) status have distinct epidemiologic, etiologic, and prognostic profiles [6, 29–35]. Given this, there is growing recognition among researchers and practitioners that breast cancer subtypes should be considered separate diseases [29, 31, 34]. Many commonly recognized behavioral and reproductive risk factors for breast cancer are associated with Luminal A and other HR + subtypes, but not with the more deadly TNBC, which is more prevalent among Black females [6, 12, 29–35]. Identifying and intervening on potential structural and institutional drivers of the TNBC subtype is an urgent priority for achieving health equity [12, 30, 33].

Therefore, we aimed to characterize the relationship between residence in neighborhoods with high home mortgage discrimination, a novel measure of institutional discrimination, and incidence of TNBC—and as a comparison, Luminal A breast cancer subtypes—among NH Black and NH White females in California. We estimated associations among NH Black and NH White females separately to examine how living in a community where there has been systematic exclusion of Black families from home ownership may be differentially associated with risk among these two racial/ethnic groups.

## Materials & methods

### Data

We merged 2006–2015 breast cancer case data from the California Cancer Registry (CCR), part of the Surveillance, Epidemiology, and End Results (SEER) program, with census tract level measures based on 2007–2013 data from the Home Mortgage Disclosure Act (HMDA) database, 2007–2011 American Community Survey (ACS) five-year estimates [5], and population estimates based on the 2010 US Census [36].

#### Breast cancer cases

Case data are from the California Cancer Registry (CCR, http://ccr.ca.gov/), a complete population-based repository containing detailed demographic, tumor, treatment, and survival information for all new cancer cases since 1988 (> 3.5 million). The CCR comprises three of the National Cancer Institute’s Surveillance, Epidemiology, and End Results (SEER) program registries (seer.cancer.gov/about). CCR data, derived primarily from the patient’s medical record, included in this analysis were: age, race/ethnicity, 2010 census tract identifiers of residential address at diagnosis, and tumor HR (estrogen and progesterone receptor) and HER2Neu status, which were used to classify breast cancer subtype. Given the focus on understanding whether institutional racism is uniquely associated with triple negative breast cancer, the analysis was restricted to TNBC (HR-/HER2-) and, as a comparison, Luminal A (HR + /HER2-) subtypes.

All NH Black and NH White female cases of primary breast cancer aged 20 + and diagnosed in California between January 2006 and December 2015 were eligible for inclusion (NH Black *n* = 19,563, NH White *n* = 185,082). We excluded cases of in-situ breast cancer, any cases that were not diagnosed with TNBC or Luminal A subtypes (including those for whom tumor subtype was not known), cases with a residential address unknown or not able to be geocoded, and cases residing outside of California metropolitan statistical areas (MSAs), as the racial bias index could only be calculated in metropolitan areas. After exclusions, the final sample consisted of *n* = 11,063 NH Black and *n* = 107,318 NH White cases of breast cancer over the study period. This research was covered under the Greater Bay Area Cancer Registry (GBACR) IRB protocol # 18-24619 at the University of California, San Francisco. The GBACR IRB approval covers secondary analyses of de-identified cancer registry data without informed consent.

#### California metropolitan census tracts

Of the 8,057 census tracts in California, 7,836 with their centroid within an MSA were included in the analysis. Data at the census tract level included a racial bias index [10], neighborhood stability, and neighborhood socioeconomic status (SES) [37], detailed below.

#### Racial bias index

Racial (anti-Black) bias in mortgage lending was estimated using 2007–2013 data from the Home Mortgage Disclosure Act (HMDA) database. Data were accessed from the Federal Financial Institutions Examination Council (FFIEC) HMDA website (http://www.ffiec.gov/hmda/).

We used a previously developed racial bias in mortgage lending index based on the HMDA data [10]. The index is described in detail elsewhere [10]. In brief, continuous surface maps were created for each MSA by interpolating the point estimates of the odds of home mortgage denial for a NH Black applicant as compared to a NH White applicant, adjusting for applicant sex and the income-to-loan ratio. These values were then averaged for each census tract to produce the “racial bias index,” which was collapsed into quintiles for the analysis. Values > 1.0 indicate greater odds of denial for NH Black versus NH White applicants for the given census tract.

#### Covariates

Associations between racial bias in home mortgage lending and breast cancer incidence could be confounded by the socioeconomic resources of a community. Therefore, we included the Yang Neighborhood SES Index [38], an adaptation of the Yost Index for American Community Survey (ACS) data [37]. This is a composite of seven neighborhood indicator variables derived from the 2007–2011 ACS: (1) education index (median school years, percentage of high school graduates); (2) proportion with a blue collar job; (3) proportion older than 16 in the workforce without a job; (4) median household income; (5) proportion below 200% of the poverty level; (6) median rent; (7) median house value [36, 38]. The neighborhood SES index was mean-standardized and included in models to adjust for confounding. As a measure of neighborhood stability, we adjusted for the percent of residents in a census tract who reported living in the same house one year ago on the 2007–2011 American Community Survey [37].

### Statistical analysis

We used Poisson regression with generalized estimating equations (GEE) to estimate incidence rate ratios (IRRs) and 95% confidence intervals (95% CIs) for associations between racial bias in mortgage lending and census tract-, race/ethnicity-, and age group-specific case counts, with the 2010 population count (times ten) as the off-set term [36]. We used an autoregressive (lag 1) [AR(1)] correlation structure to account for clustering by census tract. As a sensitivity check, we also ran Poisson models with exchangeable and independent correlation structures. Results were similar, so only the AR(1) results are reported. Model 1 adjusts for age (quadratic transformation of mid-point of each age group). Model 2 adjusts for hypothesized confounders, neighborhood SES (mean-standardized) and neighborhood stability (continuous). The estimation of the racial bias index was implemented in R and all other statistical analyses was performed using SAS v9.4.

## Results

Table 1 displays descriptive statistics for California metropolitan census tracts. The racial bias index was skewed right and ranged from 0.3 to 86.2 with a median of 2.2.

**Table 1:** Characteristics of census tracts in California MSAs (*n* = 7,836)

**Census tract characteristics**	**Range**	**Mean (SD)**	**Median (IQR)**
**Racial bias index (*n* = 7,836)**^**a**^	0.3, 86.2	2.8 (2.6)	2.2 (1.5, 3.4)
Q1	0.3, 1.3	1.0 (0.2)	1.0 (0.8, 1.2)
Q2	1.3, 1.9	1.6 (0.2)	1.6 (1.5, 1.7)
Q3	1.9, 2.5	2.2 (0.2)	2.2 (2.0, 2.3)
Q4	2.5, 3.8	3.1 (0.4)	3.0 (2.7, 3.3)
Q5	3.8, 86.2	6.2 (4.1)	5.1 (4.3, 6.6)
**SES index (*n* = 7,775)**^**b**^	− 6.2, 3.1	0.0 (1.0)	0.0 (− 0.8, 0.8)
**Neighborhood stability (*n* = 7,805)**^c^	8.1, 100	84.2 (9.5)	85.9 (79.9, 90.6)

^a^Racial Bias Index = census-tract average odds of a mortgage denial for a Black applicant as compared to a White applicant, adjusting for individual sex, and the ratio of the loan amount to the applicant's gross annual income (data from Home Mortgage Disclosure Act, 2007–2013 (https://www.ffiec.gov/hmda/))

^b^Yang Neighborhood SES Index = validated, census-tract level composite of median school years, percentage of high school graduates, proportion with a blue collar job, proportion older than 16 in the workforce without a job, median household income, proportion below 200% of the poverty level, median rent, and median house value, derived from 2007 to 2011 American Community Survey [36, 37]

^c^Neighborhood stability = census tract percent of residents who lived the same house one year ago on 2007–2011 American Community Survey [36]

Abbreviations: *SES* socioeconomic status, *SD* standard deviation, *IQR* interquartile range

Table 2 displays the characteristics of cases included in the analysis (*n* = 118,381), overall and disaggregated by race/ethnicity and subtype. Relative to NH Whites, NH Black females with breast cancer had a higher proportion of TNBC (26.2% of NH Black vs 11.8% of NH White cases) and a lower proportion of Luminal A subtypes (73.8% of NH Black vs 88.2% of NH White cases). Among both racial/ethnic groups, those with TNBC were on average younger than those with Luminal A subtypes. Relative to NH White females, NH Black females also resided in census tracts with lower socioeconomic status, less neighborhood stability, and less racial bias in home mortgage lending; however, within-race/ethnicity differences in census tract characteristics between the two subtypes were minimal.

**Table 2:** Age of primary breast cancer cases and characteristics of census tracts where they resided at time of diagnosis, by race/ethnicity and subtype, 2006–2015 (*n* = 118,381)

	Overall			NH Black			NH White		
	Both subtypes (*n*=118,381)	Luminal A (*n*=102,853 (86.9%))	TNBC (*n*=15,528 (13.1%))	Both subtypes (*n*=11,063)	Luminal A (*n*=8,170 (73.8%))	TNBC (*n*=2,893 (26.2%))	Both subtypes (*n*=107,318)	Luminal A (*n*=94,683 (88.2%))	TNBC (*n*=12,635 (11.8%))
Age (median (IQR))	64 (54, 73)	64 (54, 74)	60 (51, 71)	60 (51, 70)	61 (52, 71)	58 (49, 67)	64 (54, 74)	65 (55, 74)	61 (51, 71)
Racial bias index (median (IQR))^a^	2.2 (1.5, 3.4)	2.2 (1.5, 3.5)	2.2 (1.5, 3.4)	2.0 (1.4, 2.9)	2.0 (1.4, 2.9)	2.0 (1.4, 3.0)	2.3 (1.5, 3.5)	2.3 (1.5, 3.5)	2.2 (1.5, 3.5)
Q1 (%)	17.7	17.7	18.2	22.0	22.1	21.7	17.3	17.3	17.3
Q2 (%)	19.9	19.8	20.5	23.0	22.9	23.2	19.5	19.5	19.9
Q3 (%)	20.2	20.1	20.8	21.5	21.4	21.6	20.0	20.0	20.6
Q4 (%)	21.0	21.1	20.5	19.2	19.1	19.4	21.2	21.3	20.8
Q5 (%)	21.2	21.4	20.0	14.3	14.4	14.1	21.9	22.0	21.4
SES index (median (IQR))^b^	0.5 (−0.2, 1.1)	0.5 (−0.2, 1.1)	0.3 (−0.4, 1.0)	−0.4 (−1.0, 0.3)	−0.4 (−1.0, 0.4)	−0.4 (−1.0, 0.3)	0.6 (−0.1, 1.2)	0.6 (−0.1, 1.2)	0.5 (−0.2, 1.1)
Neighborhood stability (median (IQR))^c^	86.4 (81.0, 90.7)	86.5 (81.0, 90.8)	86.1 (80.5, 90.5)	85.7 (79.9, 90.3)	85.8 (80.0, 90.5)	85.4 (79.5, 90.1)	86.5 (81.1, 90.8)	86.6 (81.1, 90.8)	86.3 (80.8, 90.5)

Data restricted to invasive breast cancer cases for whom census tract and tumor subtype information were known (Luminal A or TNBC), and who resided in California MSAs at time of diagnosis (*n* = 118,381)

^a^Racial Bias Index = census-tract average odds of a mortgage denial for a Black applicant as compared to a White applicant, adjusting for individual sex, and the ratio of the loan amount to the applicant's gross annual income (data from Home Mortgage Disclosure Act, 2007–2013. (https://www.ffiec.gov/hmda/)); Q1: [0.3, 1.3), Q2: [1.3, 1.9), Q3: [1.9, 2.5), Q4: [2.5, 3.8), Q5: [3.8, 86.2]

^b^Yang Neighborhood SES Index = validated, census-tract level composite of median school years, percentage of high school graduates, proportion with a blue collar job, proportion older than 16 in the workforce without a job, median household income, proportion below 200% of the poverty level, median rent, and median house value, derived from 2007 to 2011 American Community Survey [5, 36, 37]

^c^Neighborhood stability = census tract percent of residents who lived the same house one year ago on 2007–2011 American Community Survey [37]

Abbreviations: *HR* hormone receptor status, *HER2* human epidermal growth factor receptor, *SES* socioeconomic status, *SD* standard deviation, *IQR* interquartile range

Table 3 shows the results of the Poisson regression estimating the association between racial bias in mortgage lending and breast cancer incidence. Model 1 shows a modest and positive association between racial bias and rates of Luminal A breast cancer among NH White females (Q5 IRR = 1.05, 95% CI = 1.02, 1.07), which is attenuated by the inclusion of neighborhood confounders in Model 2 (Q5 IRR_adj_ = 1.02, 95% CI = 0.99, 1.04). No association was found between racial bias and incidence of Luminal A breast cancer among NH Black females (Q5 IRR_adj_ = 0.92, 95% CI = 0.85, 1.00), nor between racial bias and TNBC among either racial/ethnic group (NHB: Q5 IRR_adj_ = 0.94, 95% CI = 0.83, 1.07; NHW: Q5 IRR_adj_ = 1.01, 95% CI = 0.95, 1.07).

**Table 3:** Incidence rate ratios (IRRs) and 95% confidence intervals (CIs) describing association between racial bias in mortgage lending and incidence of breast cancer in California metropolitan areas from 2006 to 2015, by race/ethnicity and subtype (*n* = 118,381)

	**Non-Hispanic Black**		**Non-Hispanic White**	
	Luminal A (*n* = 8,170)	TNBC (*n* = 2,893)	Luminal A (*n* = 94,683)	TNBC (*n* = 12,635)
	IRR (95% CI)	IRR (95% CI)	IRR (95% CI)	IRR (95% CI)
**Model 1**^**a**^				
Racial bias index Q1	REF	REF	REF	REF
Q2	1.02 (0.94, 1.10)	1.03 (0.92, 1.15)	0.99 (0.96, 1.02)	1.01 (0.95, 1.07)
Q3	1.03 (0.95, 1.11)	1.04 (0.93, 1.16)	1.01 (0.98, 1.04)	1.03 (0.97, 1.09)
Q4	1.05 (0.97, 1.13)	1.08 (0.96, 1.21)	1.03 (1.00, 1.06)	1.00 (0.94, 1.06)
Q5	0.95 (0.88, 1.03)	0.95 (0.84, 1.08)	1.05 (1.02, 1.07)	1.01 (0.96, 1.07)
**Model 2**^**b**^				
Racial bias index Q1	REF	REF	REF	REF
Q2	1.00 (0.93, 1.07)	1.02 (0.91, 1.15)	0.99 (0.96, 1.02)	1.01 (0.95, 1.07)
Q3	1.00 (0.93, 1.08)	1.03 (0.92, 1.16)	1.00 (0.97, 1.02)	1.03 (0.97, 1.09)
Q4	1.01 (0.93, 1.09)	1.07 (0.95, 1.20)	1.01 (0.98, 2.03)	0.99 (0.94, 1.05)
Q5	0.92 (0.85, 1.00)	0.94 (0.83, 1.07)	1.02 (0.99, 1.04)	1.01 (0.95, 1.07)

Data restricted to invasive cases for whom census tract and tumor subtype information were known (Luminal A or TNBC), and who resided in California MSAs at time of diagnosis (*n* = 118,381)

Racial Bias Index = census-tract average odds of a mortgage denial for a Black applicant as compared to a White applicant, adjusting for individual sex, and the ratio of the loan amount to the applicant's gross annual income (data from Home Mortgage Disclosure Act, 2007–2013 (https://www.ffiec.gov/hmda/)); Q1: [0.3, 1.3), Q2: [1.3, 1.9), Q3: [1.9, 2.5), Q4: [2.5, 3.8), Q5: [3.8, 86.2]

^a^Model 1: Poisson regression model with census tract-, race/ethnicity-, and age group-specific case counts, with log of the population as the off-set term, and using generalized estimating equations with an AR(1) correlation structure with clustering by census tract, adjusted for age and age^^2^

^b^Model 2: Same as Model 1, but also adjusted for the following census tract-level variables modeled continuously: Yang Neighborhood SES Index (validated, census-tract level composite of median school years, percentage of high school graduates, proportion with a blue collar job, proportion older than 16 in the workforce without a job, median household income, proportion below 200% of the poverty level, median rent, and median house value) [36, 37] and neighborhood stability (census tract percent of residents who lived the same house one year ago on 2007–2011 American Community Survey) [5]

All census tract data are from the 2010 US Census, [36] 2007–2011 American Community Survey [37], and 2007–2013 Home Mortgage Disclosure Act (https://www.ffiec.gov/hmda/)

Abbreviations: *IRR* incidence rate ratio, *CI* confidence interval, *TNBC* triple negative breast cancer

## Discussion

This is the first study, to our knowledge, to explore whether racial discrimination in home mortgage lending—a key measure of institutional racism—is associated with breast cancer incidence. We estimated incidence of TNBC and Luminal A separately, given these two subtypes are etiologically and epidemiologically distinct, and in response to calls to better understand the potential structural drivers of aggressive breast cancer subtypes (like TNBC) among Black females [12, 33]. Consistent with prior literature, we found the TNBC subtype to be more prevalent among NH Black females with breast cancer, relative to NH Whites; and for both racial/ethnic groups, those presenting with TNBC were younger than those presenting with Luminal A breast cancer [5]. Our primary analysis revealed that census tract level racial bias in home mortgage lending, linked with breast cancer cases at the time of diagnosis, was not associated with incidence of Luminal A or TNBC subtypes among NH Black or NH White females in California. There are several explanations for the null associations we observed.

First, the degree of protective versus harmful features of environments with higher racial bias in home mortgage lending may neutralize risk for breast cancer. On the one hand, communities with more housing discrimination, and the individuals who live in them, may possess more structural advantages and health-promoting resources. This interpretation is consistent with our data showing that census tracts with more racial bias in mortgage lending had higher socioeconomic status (median neighborhood SES in racial bias Q1 =  − 0.3; in Q5 = 0.2). Previous studies using HMDA data showed protective associations of housing discrimination with breast cancer mortality and other adverse health outcomes [23–25]. For example, Collin et al. (2020) found that females with breast cancer who lived in Georgia census tracts with greater racial bias in home mortgage lending had improved breast cancer survival compared to those in lower bias tracts [25]. This finding aligns with studies showing protective associations between racial bias in home mortgage lending with birth outcomes [24] and general health status [23]. Communities with greater levels of racial bias in home mortgage lending may have a greater concentration of socioeconomic and health-promoting resources and opportunities due to systematic investment [23, 25]. In addition, there may be a selection effect in which individuals who are able to live in these exclusionary communities may possess more socioeconomic resources and greater means to access improved health [23, 24].

On the other hand, communities with more housing discrimination may be characterized by toxic levels of psychosocial stress, in addition to other exclusionary policies and practices which could harm the health of all community-members, and of Black residents in particular [10, 13, 21, 25]. This interpretation is consistent with several studies in the Milwaukee, Wisconsin area, which found that racial bias in home mortgage lending was associated with higher rates of all-cause mortality among Black individuals living with breast cancer [10] and colorectal cancer [21]. In short, communities with higher levels of racial bias in home mortgage lending may be characterized by both health-promoting resources, and health-damaging exposures; these conflicting forces may neutralize to produce the null associations we observed.

Another plausible explanation for our findings is that racial bias in mortgage lending measured at the time of diagnosis is not etiologically relevant for the development of breast cancer. Each of the prior studies that observed harmful or protective effects of housing discrimination on health examined outcomes which may be more sensitive to the current social and environmental context, including mortality among those living with cancer [10, 21, 25], birth outcomes [24], and general health status [23]. Breast cancer has a long latency period, with risk factors accumulating across the life course and at key developmental stages [39–41]. Hence, linking neighborhood factors to individuals’ addresses at the time of diagnosis limited our ability to measure the neighborhood context earlier in life, during a potentially more salient etiologic window for the development of breast cancer [39, 40, 42]. While we adjusted for neighborhood residential stability in our models, the only data available was based on mobility from the past year, which does not account for all movement across the lives of individuals in our sample. Future longitudinal studies incorporating detailed residential history data with measures of racial bias and other forms of institutional racism could elucidate how structural conditions early in life are associated with breast cancer risk in adulthood, and inform targeted structural interventions during key etiologic windows [40].

This study had several strengths. First, by leveraging data from the HMDA, we were able to measure one type of institutional racism directly, rather than the more common approach of measuring racial residential segregation as a proxy for discriminatory practices [21]. The explicit examination of institutional racial discrimination, rather than its consequence, is important for illuminating injustice, increasing accountability, and informing policy interventions [10, 21, 25]. Second, compared to cancer registries in other states, the CCR is distinguished by collecting HER2Neu information starting in the mid-2000s, thus allowing for more granular subtype ascertainment over a longer period of time. Finally, our analytic design allowed us to estimate subtype-specific incidence of breast cancer. Several previous case-only studies demonstrated associations between racism-related exposures and odds of having a HR- versus HR + subtype among females living with breast cancer [28, 43, 44]. However, by restricting the analysis to those diagnosed with breast cancer, the case-only design cannot estimate incidence [45]. In contrast, the use of a population denominator allowed us to directly estimate associations between census tract characteristics and incidence rate ratios for Luminal A versus TNBC subtypes, a novel contribution to the best of our knowledge.

This study also had several limitations. The measurement of census tract characteristics at the time of breast cancer diagnosis, rather than earlier in the life course, may have limited utility for identifying neighborhood determinants of breast cancer risk. The analysis was restricted to California metropolitan statistical areas and results are not intended to generalize to more rural parts of California, nor to other states. In addition, census tracts are an imperfect proxy for neighborhoods and previous work found associations between neighborhood factors and cancer incidence differed by geographic unit of analysis [19]. The racial bias index does not capture discrimination against renters, potentially under-estimating the extent of institutional racism [21, 25]. Moreover, the racial bias index was averaged from 2007 to 2013, which may mask changing dynamics of segregation and gentrification over the study period. Finally, while we conceptualized neighborhood socioeconomic status and residential mobility as potential confounders of the association between racial bias and breast cancer risk, they could also be on the causal pathway. We are unable to formally evaluate mediation using cross-sectional data; however, the similar pattern of results from our adjusted and unadjusted models suggests that any confounding or mediation by these factors was minimal. Relatedly, the CCR data lack potentially important individual-level socioeconomic, behavioral, and psychosocial factors which may mediate or moderate associations between neighborhood context and breast cancer incidence; exploring these mechanisms is an important direction for future research.

## Conclusion

The explicit measurement of home mortgage discrimination and other forms of institutional racism in cancer research is a critical step toward identifying structural determinants, holding individuals and institutions accountable, and informing policy change. We applied a novel measure of institutional racism derived from publicly available data, which had previously been associated with survival outcomes among those living with cancer, to subtype-specific breast cancer incidence. Our null findings of association between racial bias in home mortgage lending and incidence of Luminal A or TNBC may be due to neutralizing harmful and health-promoting forces, or due to the limitations and timing of the data. We caution against an interpretation of these findings that home mortgage discrimination does not increase breast cancer risk, but rather call for more research linking these and other measures of institutional racism in early life with the progression of breast cancer risk across the life course.

## Data Availability

California Cancer Registry data are confidential.
